# Self-Reported Sleep Duration Is a Useful Tool to Predict Sarcopenia in Chilean Older Adults: Evidence from the ALEXANDROS Longitudinal Study

**DOI:** 10.3390/jpm14060578

**Published:** 2024-05-28

**Authors:** Myriam Gutiérrez, Carlos Márquez, Lydia Lera, Patricio Peirano, Felipe Salech, Cecilia Albala

**Affiliations:** 1 Aging, Age and Quality of Life Nucleus, Public Nutrition Unit, Institute of Nutrition and Food Technology (INTA), University of Chile, Santiago 7830490, Chile; 2 Healthy Brain Unit, Neurology and Neurosurgery Northern Department, University of Chile Clinical Hospital, Santiago 8380456, Chile; 3 Núcleo Magíster en Salud de la Mujer (MSM), Facultad de Medicina y Ciencias de la Salud, Universidad Mayor, Santiago 7500994, Chile; 4 Internal Medicine Department, Universidad de La Frontera, Temuco 4811230, Chile; 5 Latin Division, Keiser University Campus, Fort Lauderdale, FL 33409, USA; 6 Sleep and Functional Neurobiology Laboratory, Human Nutrition Unit, Institute of Nutrition and Food Technology (INTA), University of Chile, Santiago 7830490, Chile; ppeirano@inta.uchile.cl; 7 Falls and Fracture Clinic, Geriatrics Section, Advanced Clinical Research Center (CICA), University of Chile Clinical Hospital, Santiago 8380456, Chile

**Keywords:** aging, skeletal muscle, sleep patterns, cohort, Latin America

## Abstract

Age-related sleep disorders share common pathways with sarcopenia. Prospective data from Latin American populations are scarce, and the association between sleep disorders and sarcopenia in Chileans remains unknown. Thus, we aimed to study the longitudinal association between sleep disorders and sarcopenia in a cohort study of 1116 community-dwelling Chilean older people ≥60 years old from the ALEXANDROS cohorts. After the exclusion criteria, 318 subjects were followed. Sociodemographic data, self-reported chronic diseases, sedentarism, sleep characteristics, anthropometric measurements, handgrip strength, and muscle performance were assessed. Results indicated that at baseline, the prevalence of sarcopenia was 24.10% without gender differences, and the prevalence of self-reported sleep problems was 23.3%, higher in women (26.46% versus 17.15% in men). The adjusted Cox regression models for sarcopenia showed an association between sarcopenia, sleep disorders (HR = 2.08, 95% IC 1.14–3.80), and long sleep duration (HR = 2.42, 95% IC 1.20–4.91). After 8.24 years of follow-up, there were 2.2 cases of sarcopenia per 100 person-years. This study demonstrates that sleep disorders are an independent risk factor for sarcopenia in Chilean older people. The identification of sleep disorders through self-reported data provides an opportunity for early identification of risk and cost-effective sarcopenia prevention.

## 1. Introduction

Aging is a complex, multifactorial process with physiological changes across multiple systems, including the musculoskeletal system [1] and sleep architecture [2]. There is increasing interest in understanding the relationship between age-related skeletal changes and sleep patterns [3]. However, longitudinal evidence from Latin American populations is scarce, and it remains unknown whether there is an association between sleep duration and sarcopenia in older Chileans.

Sarcopenia is a progressive loss of muscle mass [4] coupled with a decrease in skeletal muscle strength, quality, or function [5]. It is associated with impaired physical function [6], falls [7], and fractures [8] and predicts all-cause mortality in older Chileans [9]. For this reason, the early identification of risk factors for sarcopenia prevention is crucial.

Sleep disorders, including the disruption of circadian rhythms, have been associated with the onset and progression of several dysfunctions, including sarcopenia. In the multifactorial pathophysiology of sarcopenia, there are common anabolic and catabolic pathways involved in both muscle balance and sleep disorders [10]. Age-associated changes in sleep patterns [11] include clinical manifestations such as difficulty in falling asleep, less deep sleep, shorter sleep duration, reduced sleep quality, greater number and duration of awakening episodes, earlier awakening, and increased daytime somnolence, among others [2,12]. Mechanisms behind this association include nutritional and metabolic interference [13], pro-inflammatory markers [14], and molecular [15], behavioral [15], and endocrine [16] factors shared with sarcopenia.

Previous studies in Asian populations [17,18] have shown a U-shaped association between sarcopenia and sleep duration in older people, with a higher incidence of sarcopenia in people with short and long sleep duration [19], especially in women [17]. In Latin American populations, cross-sectional analysis has shown an association between the risk of sleep disorders such as sleep apnea and low muscle mass in Brazilian older people [20]. In Chile, the evidence in this area is exiguous. A cross-sectional study found that Chilean older people with more than two nocturnal awakenings or short sleep duration had limited functional physical performance [12], but the association of these variables with the incidence of sarcopenia has not been investigated. Thus far, limited prospective data are available on the relationship between sleep and sarcopenia. Nevertheless, it is still unknown whether there is an association between sleep and sarcopenia in Chilean populations, and there is no longitudinal evidence of the incidence of sarcopenia and sleep disorders in older Chileans. This research aimed to study the association between sleep disorders and the incidence of sarcopenia in community-dwelling older Chileans.

## 2. Materials and Methods

### 2.1. Setting and Sample

Data from the follow-up from the ALEXANDROS multi-cohort study [21], comprising an older population-based sample, derived from a probabilistic and representative selection of participants aged 60 years and older Chileans. Subjects were recruited from 28 randomly chosen public primary care centers in the Greater Santiago region, as described in the original protocol [21]. The sample included 1116 community-dwelling Chileans aged 60 years and above residing in Santiago. After approval by the Institutional Scientific Ethics Review Board of the Institute of Nutrition and Food Technology (INTA) of the University of Chile (Acta Nr. 23, 2012, Fondecyt 1130947), all subjects signed an informed consent form. Face-to-face interviews were conducted in 2000, 2005, 2008, and 2015 to register sociodemographic data, functional status, medical conditions, chronic diseases, sedentarism, medications in use, and sleep characteristics. Inclusion criteria considered Chilean community-dwelling older people 60 years and older with permanent residency in Santiago. An exclusion criterion was that the participants had a complete sleep evaluation at baseline and follow-up. All self-reported, anthropometric, and physical assessments were performed by trained professionals. After exclusion criteria, 318 subjects were followed for a median of 8.5 person/years of follow-up since baseline.

### 2.2. Variables

Sarcopenia was defined using the first version of the European Working Group in Older People (EWGSOP1) algorithm [4] standardized for the Chilean population [22]. The EWGSOP1 recommended using the presence of both low muscle mass and low muscle function for the diagnosis of sarcopenia [4]. For this purpose, physical and anthropometric measurements were performed. Anthropometric measurements included knee height, calf circumference, and hip circumference for the estimation of muscle mass. Muscle mass was estimated using the appendicular skeletal muscle mass (ASM) prediction equation based on anthropometric measurements [23]. For Chilean older people, the ASM vas previously validated and correlated with dual-energy X-ray absorptiometry (DEXA) [24]: ASM (kg) = 0.107 (weight in kg) + 0.251 (knee height in cm) + 0.197 (calf circumference in cm) + 0.047 (dynamometry in kg) − 0.034 (hip circumference in cm) + 3.417 (sex male) − 0.020 (age in years) − 7.646; and the estimated cut-off points for reduced muscle mass were ≤7.45 kg/m^2^ in men and ≤5.88 kg/m^2^ in women [24]. For the assessment of low muscle function, we measured both muscle strength and muscle performance. Muscle strength was measured with a handgrip dynamometer with reference values of ≤27 kg in men and ≤15 kg in Chilean older women [25]. This evaluation was performed with a dynamometer (JAMAR ^®^ hydraulic dynamometer and T-18 Country Technology Inc. hand dynamometer, Gays Mills, WI, USA) with an accuracy of 0.1 kg, using the dominant hand. The measurement was made with the subject in a seated position, adjusting the handle until the subject’s fingers were in a plane perpendicular to the plane of the scale (handgrip reading manometer), asking the subject to exert the maximum possible force with his or her hand. Two measurements were taken, and the higher mark was recorded, in line with previous protocols [26]. Finally, performance was assessed with the walking speed test, considering low performance as <0.8 m/s according to EWGSOP1 [4], and the five sit-to-stand test, where low performance was defined as >9 s, according to Chilean population reference values [27], to determine the severity of sarcopenia [4].

Sleep patterns were measured with a standardized self-reported questionnaire, previously validated for the population of interest [21,28]. Sleep duration was determined by the reported total night sleep time (TST) in minutes, calculated as the time difference between the usual time of going to bed at night, minus the time it takes to fall asleep, and the usual time of waking up in the morning. Short and long sleep duration was categorized as a sleep disorder if TST was ≤5 and TST was ≥9 h, respectively, as reported in previous studies [18,29,30].

Sleep disorders were defined as having at least one alteration in sleep characteristics present. The number of reported nighttime awakenings ≥1 was assessed in the question, “How many times do you wake up during the night?”. Difficulty falling asleep (more than 30 min) was assessed in the question “time it takes to fall asleep” or an affirmative answer to the question, “Is it difficult to fall asleep again?”. Excessive daytime somnolence was measured by a modified version of the Epworth Sleepiness Scale [31]; the categories were dichotomized into low somnolence (answers “never falls asleep” or “rarely falls asleep”) and high somnolence (answers “falls asleep easily” or “always falls asleep”), where high somnolence was considered a disorder. On the other hand, three categories were established for nighttime sleep restorative capacity: “good” if waking up rested, “fair” if waking up partially rested, and “bad” if waking up tired, with “bad” being considered a sleep disturbance. Finally, self-reported sleep disorders were measured through the question, “Do you feel that you have disorders or problems with your sleep?” with a dichotomous response, which, if affirmative, was considered a sleep disorder as well as if they consulted a doctor for sleep disorders in those who answered yes, as described previously [21,28].

### 2.3. Covariates

Self-reported sociodemographic variables, including age, sex, education, and living alone condition, were included, given their relevance for stratification and control [32]. Behavioral variables such as the level of physical activity and smoking habits were also included. Physical activity levels were categorized as “low,” “moderate,” and “high” according to the response categories of the questionnaire. Self-reported comorbidities were reported to determine multimorbidity (2 or more diseases). Weight and height variables were used to calculate the body mass index (BMI) and determine nutritional status [33,34], according to World Health Organization (WHO) categories [35].

### 2.4. Statistical Analysis

All statistical analyses were performed using STATA 14 (Stata Statistical Software: Release 14; StataCorp, College Station, TX, USA). Preliminary analyses were performed to check the normality of the distribution of the data. The continuous variables were expressed as mean SD and 95% confidence intervals (CI) [36]. The categorical variables were expressed as percentages and 95% CI. The difference between sexes was calculated by a 2-sample mean comparison *t*-test or Pearson chi2 test, according to the variable. Three subgroups were considered to analyze differences in the risk of sarcopenia according to sleep duration [3]: short sleep (≤5 h), normal sleep, and long sleep (≥9 h). Cox proportional hazard models were performed to estimate the adjusted risk of sarcopenia over time for people with sleep problems and according to sleep duration. Adjustment was made for age, sex, sociodemographic variables, chronic diseases, and sleep medication usage. Kaplan–Meier curves for each subgroup were built to compare the estimated and observed time of sarcopenia incidence.

## 3. Results

### 3.1. Sample Description at Baseline

The sample at baseline included 1116 participants with a mean age of 73.22 ± 8 years (66.04% female). The general characteristics of the sample are described in Table 1. At baseline, most participants attended school, with 12 years or more schooling, higher in men than in women. Men reported a better health perception, fewer comorbidities, higher paid work, higher physical activity, and less obesity than women. Most of the sample was overweight or obese.

### 3.2. Cross-Sectional Analysis at Baseline

The cross-sectional analysis stratified by sex is described in Table 2. Women reported higher use of sleep drugs, difficulty falling asleep, difficulty conciliating sleep, more frequent consults with a doctor, more medication for sleep, and less restorative nighttime sleep than men.

The cross-sectional analysis stratified by sarcopenia is described in Table 3. We found a significant association between sarcopenia, the use of sleep drugs, short sleep, and long sleep. Thirty-one percent of the study population had at least one sleep disorder, with this prevalence significantly higher in subjects with sarcopenia (39.03%) than those without sarcopenia (28.45%).

### 3.3. Longitudinal Analysis at Follow-Up

For the longitudinal analysis, after inclusion criteria, the final sample included 318 subjects that were followed from 4.96 to 14.90 years (median 8.89 years of follow-up).

The Cox regression models for sarcopenia by sleep disorders are described in Table 4. The raw risk for sarcopenia was 1.8 (HR = 1.81, 95% CI 1.21–2.14.). The adjusted model by sex and age risk was 1.96. The adjusted model by sex, age, nutritional status, sociodemographic data, sedentarism, chronic diseases (≥3), and sleep drugs showed a higher risk, almost 2.5. Likewise, Cox regression models for sarcopenia by total sleep time showed the raw risk for sarcopenia was 1.9 (HR = 1.88, 95%CI 0.69–3.86). The adjusted model by sex and age risk was 1.87. The adjusted model by sex, age, nutritional status, sociodemographic data, physical activity, chronic diseases (≥3), and sleep drugs was 2.58.

After 3349 person-years of follow-up (median 8.5 person/years), there were 75 new cases of sarcopenia, resulting in an incidence density of 2.2 new cases of sarcopenia per 100 person-years. The incidence density in subjects with sleep disorders was 10.1 new cases of sarcopenia per 100 person-years, with 23 new cases within 226.8 person-years of follow-up. In comparison, the incidence density in subjects without sleep disorders was 9.8 new cases of sarcopenia per 100 person-years, with 52 new cases during a follow-up of 529 person-years. The estimated raw relative risk of sarcopenia was 1.03. The raw relative risk of sarcopenia by sleep disorders was estimated at 1.03.

**Figure 1:**
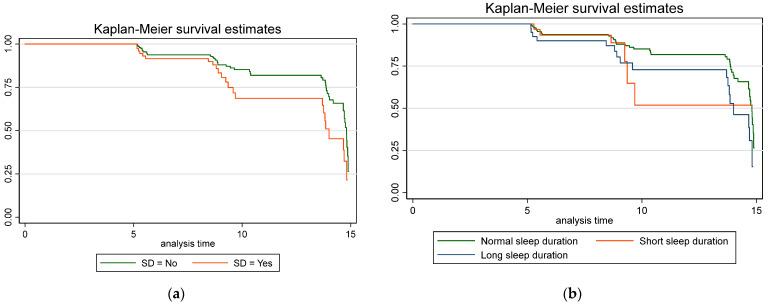
Kaplan–Meier sarcopenia estimates according to adjusted Cox proportional regression models for sleep duration and sleep disorders. (**a**) Sarcopenia estimates according to sleep duration; in blue is represented short sleep duration (≤5 h), in red is represented long sleep duration (≥9 h), and in green is represented normal sleep duration; (**b**) Sarcopenia estimates according to sleep disorders (SD); in red is indicated the presence of SF, and in blue is indicated the absence of SD.

The Kaplan–Meier survival rates for sarcopenia according to sleep duration and sleep problems were estimated, as shown in Figure 1.

## 4. Discussion

The present study shows a cross-sectional and longitudinal association between sleep disorders and sarcopenia in Chilean older people. In the baseline cross-sectional analysis, we found that sarcopenia was associated with short sleep and long sleep duration, as formerly reported as a U-shape association [3] in previous Asian cross-sectional studies [17] and other studies about sleep duration [30]. We found that the difficulty in falling asleep, the use of sleep drugs, and consulting a doctor for sleep problems were higher in females, in line with previous studies reporting differential sleep in females compared to males [37]. Moreover, recent research has shown a higher prevalence of sarcopenia in the short sleep and long sleep duration group compared with women in the normal sleep duration group, and these gender differences in sleep patterns are consistent with our findings [17,23]. 

On the other hand, taking sleep pills and consulting a doctor for sleep problems are common sleep problems that affect the quality of sleep in older people [38]. Interestingly, we also found an association between sleep drug use and sarcopenia at baseline. It has been reported that the relationship between taking sleep pills and having lower skeletal muscle mass and poorer physical performance in older people could be related to lower bioavailable hormonal levels of plasmatic testosterone [38]. Furthermore, the association between sleep architecture disturbances and sarcopenia has been linked with a pro-inflammatory status [10] and muscle catabolism via ubiquitin-proteasome and autophagy pathways [39]. These mechanisms described as implicated in sarcopenia pathogenesis have also been associated with gut microbiome dysfunctions [40]. This way, sarcopenia could be triggered by a metabolic imbalance caused by sleep disruption, with increased catabolic hormones (cortisol and myostatin) and decreased anabolic hormones (growth factor IGF-1, growth hormone, and testosterone) [10], supporting the hypothesis of a muscle secretome endocrine crosstalk [16]. 

Neural and inflammatory alterations with the participation of catecholamines and cortisol would also negatively affect the musculoskeletal system, exhibited in the activation of pathways of lower synthesis and higher protein degradation [41]. In addition, increasing evidence supports the hypothesis of the importance of sleep in muscle balance between synthesis/degradation and recovery [10]. Indeed, studies in animal models have identified that supplementation with exogenous melatonin would decrease inflammation and cell death in muscle fibers and increase the synthesis of satellite cells, promoting muscle regeneration [41].

In our study, the estimated prevalence of sarcopenia in Chilean older adults at baseline (24.10%) was higher than in previous studies in our country [22] and other populations [17]. This could be explained by the older age of the study participants at the end of the follow-up (72 years old at baseline) and the heterogeneity of diagnostics criteria among studies [9]. On the other hand, the prevalence of self-reported sleep disorders was similar to recent local studies [42]. When stratifying by sarcopenia, 31% of the study population had at least one sleep disorder, with a higher prevalence in subjects with sarcopenia. The interpretation of the link between sleep disorders and sarcopenia has contributed to the understanding of the role of peripheral clocks, such as muscle circadian clocks, in the synchronization of central clocks as in the hypothalamic suprachiasmatic nucleus [43].

In the longitudinal analysis of our research, sleep disorders and long sleep were more frequent in sarcopenic participants. The adjusted Cox regression models for sarcopenia indicated that subjects with sleep disorders had proportionally twice the risk of developing sarcopenia than those unaffected by sleep disorders. Additionally, subjects with long sleep duration also had a higher risk of developing sarcopenia (HR = 2.42, 95% IC 1.18–3.62). Long sleep showed more than twice the frequency in sarcopenic (22.67%) relative to non-sarcopenic (9.47%) participants, and 42.5% of the participants sleeping 9 or more hours developed sarcopenia. Similar results were found in older Chinese [23,30] and Korean [18] population cohorts, where long sleep would increase this risk twofold [19], using the same cut-off points [18,30].

When adjusting for age categories, the proportional risk increases. Moreover, in very old subjects (≥80), the risk of sarcopenia quadruples, as observed in previous reports [44]. 

Contrary to the cross-sectional analysis, the longitudinal analysis did not show a significant increase in the risk of sarcopenia in participants who had short sleep. The sample size could explain this difference in follow-up.

Interestingly, in both adjusted models, when analyzing by nutritional status in categories according to BMI [35], obesity is maintained as a protective factor for sarcopenia (HR 0.16, 95% CI 0.07–0.36 in model 1, and HR 0.15, 95% CI 0.06–0.35 model 2), as expected by the previous reports of our research group [21]. Although there is controversy in the literature on the use of BMI to assess nutritional status in older people [45], previous studies have highlighted the utility of BMI for assessing nutritional status [33,34], and it has been used in the Chilean older population in association with sarcopenia [46].

An important strength of this study is that it is the first longitudinal research in Chile that studied sleep and its association with sarcopenia in older people based on a population sample with an 8-year follow-up, clinical assessments performed by trained professionals, and a protocol sustained by an international consensus. Previous studies have focused on different geographic regions, especially Asian and European populations, which are different from Latin American populations. The use of validated cut-off points for the older Chilean population considers that this population differs from other populations since the validity of the definitions used for the diagnosis of sarcopenia depends on the use of reference cut-off points collected from populations of similar age, race, and ethnic composition [24]. Likewise, employing a validated self-reported instrument for sleep assessment in predicting sarcopenia is clinically relevant since the diagnosis and management of sleep problems rely primarily on clinical history [47]. This bears clinical significance and feasibility, particularly in light of the considerable time and expenses involved in conducting a comprehensive overnight sleep study in a hospital that requires specialized equipment and expertise. Similar approaches have been used to assess the association between multiple sleep disorders, the risk of obstructive sleep apnea [48], lower scores in muscle mass, and a higher risk of sarcopenia in a large sample of older Latin American participants [20].

Additional implications are the therapeutical utility of the results of this study in Chilean older people. Indeed, physical activity and exercise-based interventions could contribute to treating both sleep problems and sarcopenia [49], considering a clear association between elements critically related to sleep and the appearance of sarcopenia. An aspect to consider as a potential limitation of this study is the variation in follow-up duration among different cohorts, which was effectively addressed through the Cox regression analysis. Future clinical research has the potential to enhance these findings by incorporating objective measurements of sleep patterns for a more comprehensive understanding of the sleep–wake cycle [50]. Finally, the consensus on the definition of sarcopenia [5] continuously evolves, but its use depends on population validity [7]. In this study, we used the EWGSOP1 sarcopenia diagnosis criteria [4] since its clinical relevance due to its validation in the Chilean population [24], its relationship with functional physical performance [51], mobility [48], and its clinical value in predicting fractures and mortality in the study population [8]. 

The results of this follow-up study contribute to the understanding of sleep patterns, sleep duration in particular, as a risk factor for sarcopenia. In comparison to hospital-based night sleep recordings, the detection of sleep disorders through self-reported data offers a cost-effective opportunity for early risk identification and prevention of sarcopenia in older people.

## 5. Conclusions

This study demonstrated a cross-sectional association between long sleep and short sleep duration and sarcopenia and a longitudinal association between long sleep duration and sarcopenia. Furthermore, the use of sleep drugs, difficulty falling asleep, and long sleep duration were associated with sarcopenia in community-dwelling Chilean older people. People aged 60 years and older with at least one sleep disorder would present more than twice the risk of presenting sarcopenia, highlighting the relevance of having measures for screening sleep problems and their role in the prevention of sarcopenia.

This research presents the first contribution to the link between sleep disorders and sarcopenia in a Chilean cohort using the same self-reported validated measures since baseline. Given the population’s accelerated aging, this research contributes to public health from a preventive perspective. These findings are relevant for the development of therapeutic and preventive personalized strategies for older people.

## Figures and Tables

**Table 1:** Characteristics of the sample by sex at baseline.

Variable	Men *n* = 379	Women *n* = 737	Total *n* = 1116	*p*
Mean age ± SD	72 ± 7.3	73 ± 8.3	73 ± 8.0	CI ^1^ 72.75–73.69 ^a^
● Min	60	60	60	
● Max	94	100	100	
Age group, %				
● 60–69.9 y	47.2	39.8	42.3	0.002 ^b^
● 70–79.9 y	38.8	37.6	38.0	
● ≥80 y	14.0	22.7	19.7	
Education, %				
● 0 y	11.1	20.0	15.5	0.003 ^b^
● 1–8 y	58.3	56.8	57.6	
● ≥12 y	16.7	8.85	12.8	
Living alone, %	10.5	13.3	12.1	0.080 ^b^
Self-reported health status, %				
● Excellent, good, or very good	42.7	32.7	36.1	<0.0001 ^b^
● Regular	39.6	44.8	43.0	
● Bad	17.7	22.5	20.9	
Physical activity, %	29.6	17.9	21.9	<0.0001 ^b^
Has paid work, %	72.0	11.5	17.6	<0.0001 ^b^
Number of chronic diseases, %				
● 0–1	69.2	51.7	20.3	<0.0001 ^b^
● ≥2	30.9	48.3	79.7	
Nutritional status, %				
● Normal	27.1	21.5	23.4	0.002 ^b^
● Thin	4.0	3.17	3.5	
● Overweight	45.6	40.8	42.4	
● Obese	23.3	34.5	30.7	
Sarcopenia, %	21.9	24.4	24.1	0.217 ^b^

^1^ CI: Confidence interval. ^a^ Two-sample *t*-test with equal variances. ^b^ Pearson chi2.

**Table 2:** Sleep characteristics of the sample by sex at baseline.

Sleep Variable	Men *n* (%)	Women *n* (%)	Total *n* = 318 (%)	*p* ^1^
Sleep disorders	108 (28.50)	238 (32.29)	346 (31.00)	0.194
Total sleep time (TST) ^2^				
● Short TST	52 (13.72)	90 (12.21)	142 (12.72)	0.118
● Normal TST	278 (73.35)	517 (70.15)	795 (71.24)	
● Long TST	49 (12.93)	130 (17.64)	179 (16.04)	
Sleep drugs use	46 (12.14)	180 (24.46)	226 (20.27)	<0.0001
Difficulty in conciliating sleep	122 (32.45)	334 (45.32)	456 (40.97)	<0.001
Has consulted a doctor about sleep disorders	20 (30.77)	81 (41.03)	101 (100)	<0.0001
Shiftwork	225 (59.52)	119 (16.17)	344 (30.88)	<0.0001
Night awakenings				
● 1–2	181 (68.82)	362 (69.22)	543 (69.08)	0.910
● ≥3	82 (31.18)	161 (30.78)	243 (30.92)	
Difficulty falling asleep	95 (35.99)	252 (47.91)	347 (43.93)	0.003
Has self-reported sleep problems	65 (17.15)	195 (26.46)	260 (23.30)	0.005
Has restful sleep	285 (76.00)	496 (67.81)	781 (70.61)	0.005
High daytime somnolence	175 (48.48)	311 (44.11)	486 (45.59)	0.176

^1^ Pearson chi2. ^2^ TST: Total sleep time (sleep duration). Short sleep: TST ≤ 5 h; long sleep: TST ≥ 9 h.

**Table 3:** Association between sleep characteristics and sarcopenia at baseline.

Sleep Characteristic	No Sarcopenia *n* (%)	Sarcopenia *n* (%)	Total *n* (%)	*p* ^1^
Short TST ^2^	111 (13.11)	31 (11.52)	142 (12.72)	<0001
Long TST ^2^	108 (12.75)	71 (26.39)	179 (16.04)	<0001
Sleep drugs use	146 (17.24)	80 (29.85)	226 (20.27)	<0.0001
Difficulty falling asleep	329 (39.12)	127 (47.57)	456 (41.16)	0.015
Having nighttime awakenings	595 (70.33)	191 (71.54)	786 (70.62)	0.706
High difficulty falling back asleep	187 (31.53)	66 (35.11)	253 (32.39)	0.404
Has self-reported sleep disorder	192 (22.78)	68 (25.86)	260 (23.51)	0.304
Has consulted a doctor about sleep disorders	70 (36.08)	31 (44.29)	101 (38.26)	0.226
Has restful sleep	602 (71.41)	179 (68.06)	781 (70.61)	0.404
High daytime somnolence	215 (26.58)	79 (30.74)	294 (27.58)	0.194
Shiftwork	259 (30.61)	85 (31.72)	344 (30.88)	0.734
Snoring	522 (66.92)	150 (63.29)	672 (66.08)	0.301

^1^ Pearson chi2. ^2^ TST: Total sleep time (sleep duration). Short sleep: TST ≤ 5 h; long sleep: TST ≥ 9 h.

**Table 4:** Cox regression models for sarcopenia by sleep disorders and by sleep duration.

Variable	Sleep Disorders (SD) ^a^						Sleep Duration (TST) ^b^					
	Adjusted Model 1			Adjusted Model 2			Adjusted Model 1			Adjusted Model 2		
	HR	*p*	95% CI	HR	*p*	95% IC	HR	*p*	95% IC	HR	*p*	95% CI
Sleep disorders	1.96	0.009	1.19–3.24	2.08	0.017	1.14–3.79						
Short sleep TST							1.39	0.461	0.58–3.29	1.62	0.304	0.64–4.06
Long sleep TST							2.42	0.011	1.18–3.62	2.45	0.014	1.19–4.90
Women	0.92	0.753	0.56–1.52	0.84	0.543	0.48–1.46	0.83	0.747	0.56–1.52	0.66	0.521	0.48–1.45
Age ≥80 yr	0.54	0.084	0.27–1.08	1.11	<0.001	1.06–1.17	0.52	0.065	0.26–1.04	0.39	0.023	0.17–0.88
Obesity				0.16	<0.001	0.67–0.37				0.15	<0.001	0.07–0.36
Lives alone				1.02	0.968	0.39–2.65				0.96	0.929	0.36–2.52
Education ≥8 yr				1.62	0.113	0.89–2.95				1.65	0.104	0.90–3.00
Sedentarism				1.49	0.206	0.80–2.79				1.50	0.199	0.81–2.80
Chronic diseases ≥3				1.49	0.363	0.63–3.52				1.57	0.313	0.65–3.74
Night shift				1.26	0.493	0.65–2.45				1.25	0.514	0.64–2.43
Sleep drugs				2.00	0.025	1.09–3.66				1.97	0.029	1.07–3.60

^a^ Cox regression adjusted model for sleep disorders included the presence of at least one of the following self-reported sleep disorders: total sleep time (TST) ≤ 5 h (short sleep), TST ≥ 9 h (long sleep), nocturnal awakenings ≥1, difficulty in falling asleep; self-reported sleep disturbances, or high daytime somnolence. ^b^ Cox regression adjusted model for sleep duration, where short sleep duration is defined by TST, as described in ^a^. For both regressions, adjusted model 1 was made by sex and age, and adjusted model 2 was made by sex, age, obesity, bad health, living alone, education, sedentarism, chronic diseases, and use of sleep drugs. HR: hazard ratio; CI: confidence interval.

## Data Availability

Data supporting reported results can be found upon request to corresponding authors.
